# Chicago Society of Physicians and Surgeons

**Published:** 1875-03

**Authors:** Ralph E. Starkweather


### Reports of Societies.

### CHICAGO SOCIETY OF PHYSICIANS AND SURGEONS.

*Regular Meeting, Jan.* 11, 1875.

(Reported by RALPH E. STARKWEATHER, M.D.)

### TUMOR OF DENTINE.

Dr. John Bartlett, President, in the chair.

Dr. Andrews reported a case of unusual interest and rarity, and accompanied the same with an exhibition of a microscopical preparation of a portion of the material removed, furnished him by Dr. Curtis.

A writer in the recent edition of Holmes' Surgery cites and knows only of twelve cases of tumor of dentine. The European medical museums have a total of six specimens only. Dr. Andrews had heard of but one case in this city similar to his.

Dental tumors are of two sorts. The first is a simple deformity of the tooth, its structure not being changed. The second sort is where the teeth depart from the normal structure altogether; generally the molar teeth are the ones thus affected and deformed.

The patient, a lady of eighteen years of age, exhibited a swelling on the side of the jaw, where the wisdom tooth should have presented itself, very much as though the projecting bony tumor had been due to a simple bony periostitis. A few months after having first seen the patient, the tumor or cyst began to suppurate, and it was at once incised. Upon probing, something like dead bone was felt, and the prominent point of the bone tumor had ulcerated through the gum. The patient having been anæsthetized, a longitudinal incision was made along the gum, and ineffectual attempts made to extract a supposed tooth. At length, by using a pair of strong gouged bone forceps, a fragment was broken off.

In this manner, most of the then projecting bony tumor was removed, and carbolated lotions ordered. Eight weeks later, there had been a slight loosening of the mass, and a little elevation thereof, and under anæsthesia, by using the bone forceps, a rounded tuberous bony body was removed from the cavity where a tooth should have been.

### SARCOMA AND CARCINOMA.

Dr. Andrews made some remarks as to the clinical history of the sarcoma and carcinoma, their differential diagnosis, microscopical and physical, illustrating the same by reports of cases, and by microscopical specimens, prepared and furnished by Dr. Curtis, of carcinoma of the breast.

The patient with carcinoma mammæ has died as had been anticipated, the clinical history confirming the diagnosis and prognosis made by the microscopist.

The patient with sarcoma, the second case, is now alive after two years presence of the tumor, or its fungus, which is now of the size of a large hazel-nut, and is located on the side of the foot near the malleolus.

Prof. Atlee's treatment had been followed, that of the exhibition of Fowler's solution, and with gratifying results.

Incidentally, Dr. Andrews remarked that it seemed to him that there were more cases of cancerous growths in Northern Wisconsin than in regions south of us. That consumption and cancer often prevailed in the same climates, and were more abundant the nearer one approaches to the Eastern coast of this country.

*Histology of Carcinoma.* First, there is a fibrous frame-work, in which are impacted a quantity of small round cells as follows: bands of fibres interlace and leave lacunæ, and these are packed full of rounded cells. Certain growths or germs are transmitted to the lymphatics—these become cells and are conveyed internally; probably these spaces, or lacunæ, in which the small cells are packed, are enlargements of the lymphatics — hence open right into the absorbents, convert the lymph gland itself into cancer. A characteristic of these cells in carcinoma is that they can easily be brushed out, leaving the cavities, or lacunæ, empty. They can thus be easily conveyed through the body.

There is a certain class of tumors hard to destroy; among them the sarcoma, or flesh tumor. A few weeks ago, a woman, eighteen years of age, presented herself for treatment, upon whom there were several of these growths. They had been enlarging and increasing for upwards of two years. A year ago, two had been removed but both returned, and one became larger than it had previously been. The tumors were from the size of a hazle-nut to two inches in diameter, hard, not painful; the neighboring lymphatic glands were normal in every respect. There were two on the right thigh, but the inguinal glands were sound. There was one tumor in the left inguinal region of the abdomen, and suppurating. In every case the lymphatic glands were perfectly normal —for sarcoma does not develop in the lymphatic spaces. Its cells are outside of them—hence are not taken into the absorbents. The cells of the sarcoma adhere more closely to each other than do those of carcinoma: they are spindle shaped, or more like a fibroid tumor, so that it is sometimes hard to distinguish between sarcoma and fibroid tumors. The sarcoma, unlike the carcinoma, has no framework apart from the cells. As a general rule, a diagnosis can be made between carcinoma and sarcoma. The hope of cure by excision is greater in the latter than the former.

Dr. Hyde thought it a difficult point as to whether a fungus hæmatoides—a medullary sarcoma—might not be considered cancerous.

It was somewhat remarkable that in a recent discussion on cancer, in New York, Dr. Willard Parker had said that cancer was no longer to be considered as hereditary, that it often occurred in families with no previous history of cancer.

Dr. Curtis thought that there was a difference between sarcoma and carcinoma ; the former throughout is made up of similar tissue : the latter is made up of entirely different elements. The colloid material is a degeneration of cancerous material, just as cancer may undergo fatty degeneration ; the colloid is a transformation ; it is an exaggerated species of cancer, the cells greatly dilated. In cancer there is no shading off of the cells into the fibrous tissue. The term cancer, like that of consumption, has been abandoned by some authors ; it is too general an expression.

After further discussion by Doctors Simon, Hyde, Merriam, and Andrews, the Society adjourned.

